# Branchial Anomalies: Diagnosis and Management

**DOI:** 10.1155/2014/237015

**Published:** 2014-03-04

**Authors:** Sampath Chandra Prasad, Arun Azeez, Nikhil Dinaker Thada, Pallavi Rao, Andrea Bacciu, Kishore Chandra Prasad

**Affiliations:** ^1^Department of Otolaryngology, Head and Neck Surgery, Srinivas Institute of Medical Sciences and Research, 5-7-712/3 ASRP Street, Dongerkery, Kodialbail, Mangalore, Karnataka 575001, India; ^2^Department of Otolaryngology, Head and Neck Surgery, Kasturba Medical College, Mangalore, Karnataka, India; ^3^Department of Radiodiagnosis, Kasturba Medical College, Mangalore, Karnataka, India; ^4^Department of Clinical and Experimental Medicine, Otolaryngology Unit, University Hospital of Parma, Parma, Italy

## Abstract

*Objective*. To find out the incidence of involvement of individual arches, anatomical types of lesions, the age and sex incidence, the site and side of predilection, the common clinical features, the common investigations, treatment, and complications of the different anomalies. *Setting*. Academic Department of Otolaryngology, Head and Neck Surgery. *Design*. A 10 year retrospective study. *Participants*. 30 patients with clinically proven branchial anomalies including patients with bilateral disease totaling 34 lesions. *Main Outcome Measures*. The demographical data, clinical features, type of branchial anomalies, and the management details were recorded and analyzed. *Results and Observations*. The mean age of presentation was 18.67 years. Male to female sex ratio was 1.27 : 1 with a male preponderance. Of the 34 lesions, maximum incidence was of second arch anomalies (50%) followed by first arch. We had two cases each of third and fourth arch anomalies. Only 1 (3.3%) patients of the 30 presented with lesion at birth. The most common pathological type of lesions was fistula (58.82%) followed by cyst. 41.18% of the lesions occurred on the right side. All the patients underwent surgical excision. None of our patients had involvement of facial nerve in first branchial anomaly. All patients had tracts going superficial to the facial nerve. *Conclusion*. Confirming the extent of the tract is mandatory before any surgery as these lesions pass in relation to some of the most vital structures of the neck. Surgery should always be the treatment option. injection of dye, microscopic removal and inclusion of surrounding tissue while excising the tract leads to a decreased incidence of recurrence.

## 1. Introduction

Branchial fistulas and cysts, involving soft tissues of neck, are uncommon anomalies of embryonic development that are commonly encountered by otolaryngologists. In fact, approximately 17% of all pediatric cervical masses are due to branchial anomalies. Although branchial cleft cysts are benign, superinfection, mass effect, and surgical complications account for its morbidity. Branchial apparatus, seen in the early embryonic life, has a vital role to play in the development of head and neck structures. “Branchia” is the Greek word for gill, and the same word represents these anomalies owing to their resemblance to gills of certain species as fish. Six paired branchial arches, which appear in the fourth week of embryonic life, give rise to many structures of the head and neck. Each branchial arch consists of core of mesenchyme covered externally by ectoderm and internally by endoderm. The fifth arch disappears and the sixth arch is rudimentary. Many anomalies of the head and neck region have been attributed to the aberrant development of these structures. Depending on the anatomic location, branchial anomalies are classified into first, second, third, and fourth anomalies. The course of a particular branchial anomaly is caudal to the structures derived from the corresponding arch and dorsal to the structures that develop from the following arch. Branchial anomalies are further typed into cysts, sinuses, and fistulas. Cysts are considered to be entrapped remnants of branchial cleft or sinuses; sinuses are remnants of cleft or pouches; and fistulae result from persistence of both pouch and cleft [1]. Different anomalies of the head and neck area have been attributed to the maldevelopment of branchial apparatus. The importance of knowing the development of branchial apparatus and their anomalies is in applying the knowledge during surgery, as vital structures like facial nerve and parotid are in intimate relation with many of these anomalies. We performed a ten-year retrospective study to analyze the pathophysiology, clinical features, and management of branchial anomalies.

## 2. Materials and Methods

**Figure 1:**
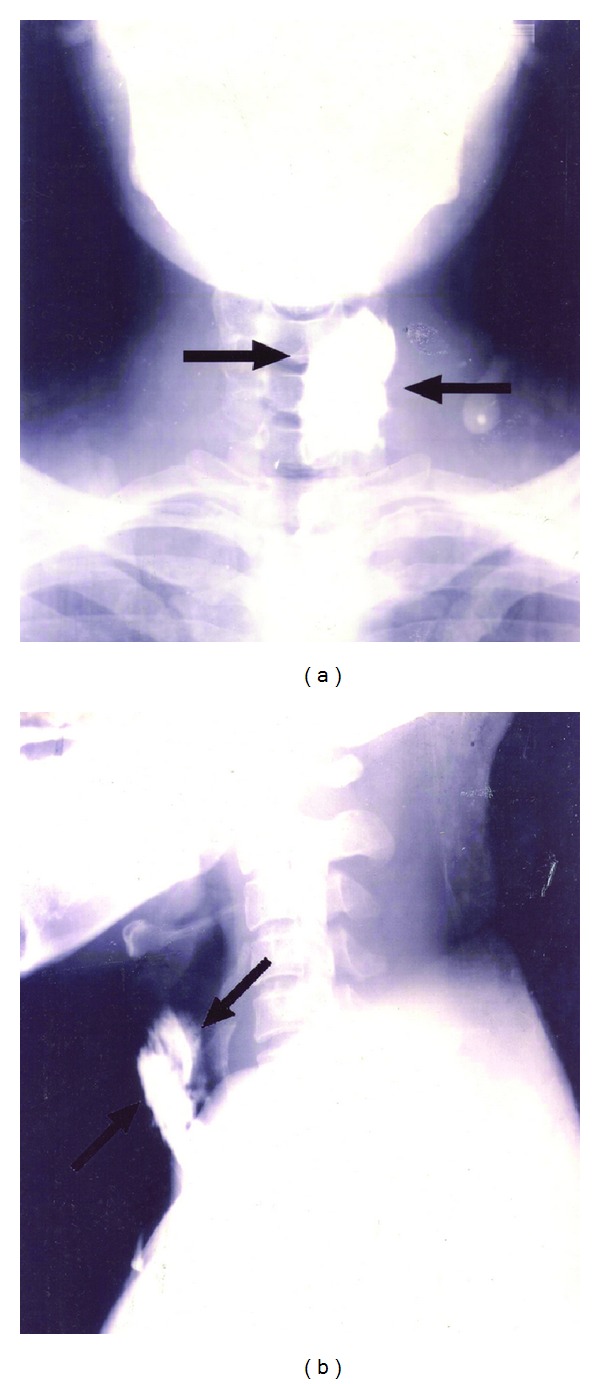
Contrast X-ray showing 4th branchial anomaly.

Ours is a retrospective study of 30 cases of branchial anomalies, which presented to the Department of Otolaryngology, Head and Neck Surgery, Kasturba Medical College, over a period of 10 years from 2000 to 2010. This study was cleared by the Manipal University Ethics Committee for Research and Publication. Age, sex, and duration of symptoms were noted from the case records. Family history and previous history of infection and/or surgery were noted. The side and site of the lesion and the site of opening of sinuses and fistula were noted. All the patients underwent routine blood examination. Patients with sinus and fistulas underwent sino-/fistulogram, by injecting contrast material urografin into the tract (Figure 1). The cystic lesions were investigated with ultrasound and CT scan. All patients were operated upon. In cases of acute infection, patients were put on intravenous antibiotics and in cases of abscess incision and drainage were done. Such patients were taken up for surgical excision of tract four weeks later. During surgery, a conscious attempt was made to remove some fascia and tissues adjacent to the branchial tracts along their path to avoid leaving behind ramifications that might lead to recurrences. The excised specimens were subjected to histopathological examination. Surgeries were performed under general anesthesia.

### 2.1. Excision of Collaural Fistula

**Figure 2:**
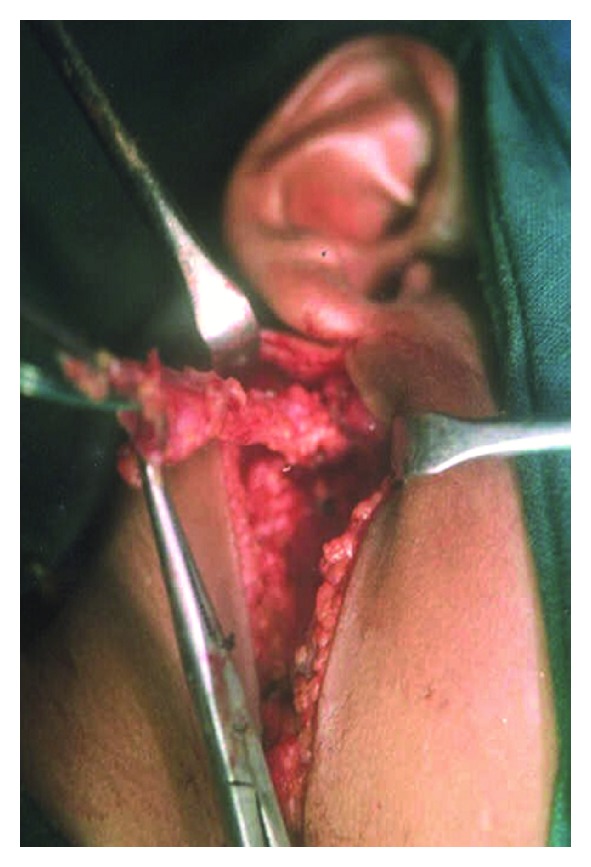
Collaural fistula.

This fistulous communication between the external auditory canal and the neck in the upper part of anterior border of sternocleidomastoid (SCM) muscle was identified by injecting methylene blue dye into the neck opening which was seen coming of an opening in the external auditory canal. A parotidectomy incision was given and facial nerve identified after superficial parotidectomy. The tract was then dissected from the surrounding tissue and followed till its opening in the external auditory canal (Figure 2). The tract was excised off its attachment to the external auditory canal and wound closed in layers.

### 2.2. Excision of Branchial Cyst

Skin incision was given over the cyst. Subplatysmal layer was elevated. SCM muscle was retracted away from the field taking care not to injure the greater auricular nerve. The cyst was carefully separated from the surrounding structures without damaging the wall. After complete excision, the wound was closed in layers.

### 2.3. Excision of Branchial Fistula

**Figure 3:**
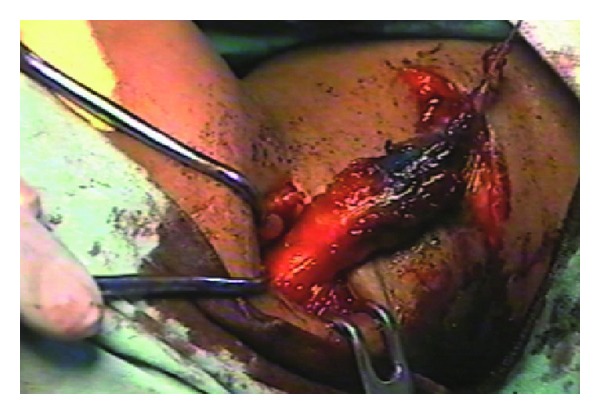
Excision of third branchial fistula.

**Figure 4:**
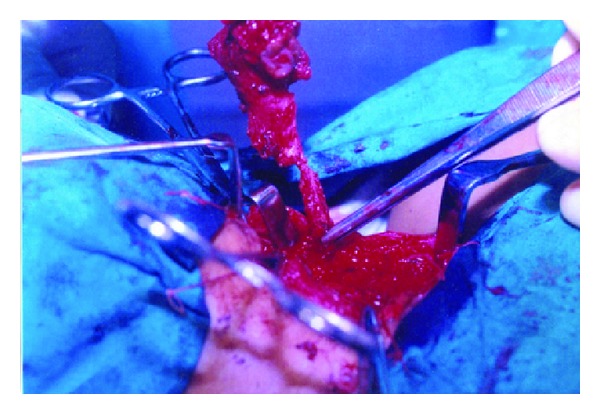
Excision of fourth branchial anomaly.

The tracts were identified by injecting methylene blue. Elliptical skin incision was made over the skin opening and the dissection proceeded in the direction of the tract. Step-ladder incision was used for the complete excision of the tract. This second incision was given at the level of the hyoid and the whole tract was brought out through this incision. It was then followed to its opening into the pharynx. During their course towards the oropharynx, the second branchial fistulae, were seen passing between the carotid bifurcations, where they were in close relation to the hypoglossal nerve. The third branchial fistulae were seen piercing the thyrohyoid membrane to open into the pyriform fossa (Figure 3). The tracts were followed to the pharynx and were excised; the pharyngeal defects were sutured. The suture lines were reinforced by a second layer of suture in the pharyngeal musculature. Wounds were closed in layers after placing a drain. The fourth branchial fistulae were seen opening into the lower part of neck near the SCM muscle. The tract then passed inferiorly into the mediastinum, looping around the arch of aorta in the left and subclavian artery in the right and back in to the neck, ascending posterior to the carotid. Then it passed between the thyroid and cricoid cartilages opening into the pyriform fossa. The tract was followed from its neck opening into the mediastinum using blunt finger dissection. The intramediastinal part of the fistulae was left behind with their ends ligated and the rest of the tract was dissected out from the superior mediastinum up to the pyriform fossa (Figure 4).

## 3. Results and Observations

Thirty patients with 34 branchial anomalies were studied retrospectively over a period of 10 years from 2000 to 2010 in the Department of Otolaryngology, Head and Neck Surgery, Kasturba Medical College, Mangalore.

### 3.1. Type of Anomalies (Table 1)

**Figure 5:**
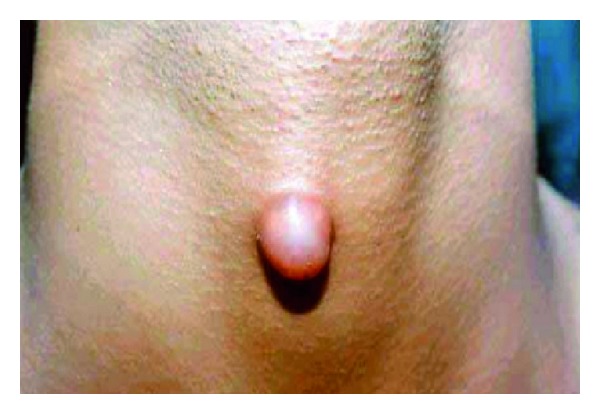
Fourth branchial anomaly.

There was maximum incidence of second branchial anomalies with 17(50%) cases. Among the first branchial anomalies, seven (20.59%) cases belonged to Work II, while six (17.65%) cases belonged to Work I (according to the Work classification). Second branchial arch anomalies were seen in 17 cases (50%). Of these, branchial cyst constituted eight (26.53%) cases while branchial fistula constituted nine (26.47%) cases. Third branchial arch anomaly was seen in two (5.88%) patients. Fourth branchial arch anomaly was seen in two (5.88%) patients (Figure 5).

Among the anatomical types of the lesion, we had a maximum incidence of fistula seen in 20 (58.82%) cases, followed by cyst in 14 (41.12%) cases.

### 3.2. Age and Sex Incidence (Table 2)

The youngest patient in our study was one and a half years and the oldest one 48 years. The mean age of presentation was 18.67 years with a standard deviation of 11.06. Only 1 (3.3%) patient of the 30 presented with lesion at birth. The remaining 29 patients (96.7%) had a late onset of the disease. The mean age of onset among this late onset group was 15.97 years.

Among the first branchial anomalies, the maximum incidence of the lesion was seen in the 11–20 age group with 5 (14.70%) cases followed by 4 (11.76%) cases in the 6–10 and 21–40 age groups. In the second arch anomalies, we had four (11.76%) cases in the 6–10 age group and seven (20.59%) cases in the 11–20 and five (14.70%) in 20–40 age groups. Considering all the anomalies together, 55.88% were males and 44.12% were females with a male to female ratio of 1.27 : 1. In the first branchial anomalies, the incidence in males was 17.65% and 20.59% in females. Among the second arch anomalies 26.47% were males, while 23.53% were females. Third and fourth anomalies were seen only in males.

### 3.3. Side Incidence

**Figure 6:**
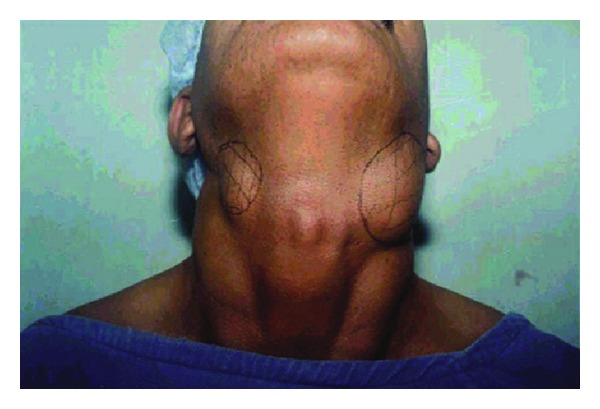
Bilateral branchial cyst.

The overall incidence of the anomalies was more on the right side (57.08%) while 42.92% of the lesions occurred on the left side. In the first arch anomalies, 8 (23.53%) cases were present on the right and 5 (14.71%) cases on the left. In the second arch anomalies 11 (32.35%) cases occurred on the right side and six (17.65%) cases on the left including one patient with bilateral branchial cysts (Figure 6). The third anomalies occurred on the right side and the fourth anomalies on the left side.

### 3.4. Clinical Features (Table 3)

In all anomalies put together, the most common clinical feature was a swelling seen in 21 (61.76) and fistula opening in 18 (52.94%) cases. Discharge from the lesion was presented by 41.58% of the patients. 26.47% of the patients had pain at the site of the lesion. Among the first arch anomaly patients, swelling in the neck and postauricular region was the most common presenting feature (29.41%). Pain and discharge were seen in 14.71%. The most common presenting feature of second branchial arch anomaly was neck swelling, seen in 26.47%, while 23.53% presented with opening in the neck. 20.59% had discharge from the lesion. Pain and fever were present in 8.82% of patients each. The third arch anomaly patient had swelling and opening in the neck along with discharge. The fourth arch anomaly patient had swelling and opening in the neck along with discharge and pain.

Fourteen patients had history of previous infection for which they had taken treatment. Five patients with second branchial arch anomalies had previous history of infection. The third and fourth arch anomaly patients also had history of previous infection.

### 3.5. Investigations

Sinogram/fistulogram was performed in all the cases. Ultrasound and CT scans were each done in 13.84% of patients. CT scan and ultrasound were done in all cases of third and fourth arch anomalies and nine cases of second arch anomaly. FNAC was done in five cases of branchial cysts.

### 3.6. Treatment (Table 4)

Acute infection was treated by a course of antibiotics in 18 (60%) cases and incision and drainage in one case (before proceeding to the excision of the lesion). All the patients underwent surgical excision of the lesion. 73.33% of the cases were managed by single incision, while 23.33% required stepladder incision.

### 3.7. Complications (Table 5)

Wound infection developed in 14.71% of the cases. Majority of this occurred in first branchial arch anomalies (11.76%). Wound gaping, which required secondary suturing, was seen in 8.82%. The recurrence rate in our series was 1.2%.

## 4. Discussion

Though described first in the early nineteenth century, the origin and classification of different branchial anomalies are highly controversial even today. The earliest description of branchial apparatus has been attributed to Von Baer in 1827. Rathke in 1828 had described the development of pharyngeal arches in the human fetus. Acherson in 1832 first recognized branchial fistula and gave branchial cyst its name. Virchow first described the branchial cleft anomalies in 1865. Cervicoaural or collaural fistula was first described by Sir James Paget in 1878. Second branchial anomalies are considered to be the commonest with figures up to 95% being reported [2]. The remainder of branchial anomalies is derived from first branchial remnants (1–8%) with third and fourth branchial anomalies being quite rare [1]. There is still a controversy regarding the origin of branchial anomalies. Several theories proposed for the development of branchial anomalies include branchial apparatus theory, cervical sinus theory, thymopharyngeal theory, and inclusion theory. Of these, the widely accepted theory is that branchial anomalies result from incomplete involution of the branchial apparatus [1].

### 4.1. Age, Sex, and Side Incidence

According to Ford et al., [3] most of the branchial anomalies arise from the second branchial cleft (92.45%). Remaining is derived from first arch remnants (4.72%) and third (1.87%) and fourth arch anomalies (0.94%) are quite rare. Bajaj et al. [4] also reported higher incidence of second branchial anomalies (78%) in their series of 80 patients. Choi and Zalzal [1] who reported a higher incidence of first branchial arch anomalies (25%) in their series still had the maximum incidence of second branchial arch anomalies (40%). In our series, we had the maximum incidence of second arch anomalies (50%) followed by first arch anomalies (38.24%). Third and fourth arch anomalies accounted 5.88% each. Cysts, sinuses, and fistulae are the three anatomical types of branchial anomalies. Choi and Zalzal [1] reported a maximum incidence of sinuses, followed by fistula. In our series cysts were the most common lesion followed by sinuses.

Though a congenital lesion, branchial anomaly usually presents late in life. The age of onset of these anomalies has been seen to vary according to the type of the lesion. Choi and Zalzal [1] have noted that mean age of presentation of cyst (18.35 years) was late compared to that of fistulae (6.28 years) and sinuses (7.82 years). This finding was confirmed in our study. In our group, it was found that fistulas (1.14 years) had an early age of onset followed by that of sinuses (4.21 years). Cysts (7.51 years) were found to have a late onset compared to the other two lesions. Ford et al. [3] have pointed out that the branchial anomalies occur more on the right side. They had 60% incidence on the right side and 40% on the left side. In the present study, we have a similar picture with right side incidence of 55.38% and left side incidence of 44.62%.

### 4.2. Clinical Features

In the study by Choi and Zalzal [1], the most common presenting features were discharge from the openings, cervical mass, and repeated infection. In our study, the most common clinical feature was a swelling seen in 21 (61.76). Discharge from the lesion was presented by 41.58% of the patients. 26.47% of the patients had pain at the site of the lesion.

### 4.3. Investigations

The diagnosis of branchial anomalies may be straightforward. However atypical lesions can be misdiagnosed. An initial correct diagnosis is crucial because experience shows that recurrence rates after surgical excision of branchial anomalies are 14% and 22% with previous infection and surgery, respectively, whereas the recurrence rate for primary lesion is 3% [5]. Although physical examination and history are the most important elements in the diagnosis, radio-diagnostic studies can add valuable information to the evaluation of a congenital neck mass. A CT scan is an accurate and noninvasive diagnostic tool, which can confirm the diagnosis or suggest an alternative diagnosis, define both the location and extent of a neck lesion, and delineate infectious process or possible malignant degeneration [6]. CT scan is useful in evaluating first branchial anomaly and the position of facial nerve. CT scan is reported to be more useful than MRI in evaluating branchial anomalies [1]. In case of sinus or fistula, sinogram or Conray contrast study can delineate the course of branchial anomaly. In the series by Choi and Zalzal [1], CT scan was performed on 15.38% of patients, sonogram/contrast study was performed on 7.69% of patients, and ultrasound/MRI was done on 1.92% of patients. In our series, intraoperative methylene blue injection was performed in all cases; CT scan was done in 10.77% of cases. Ultrasound was done in 10.77% of cases. None of the patients underwent MRI.

### 4.4. Treatment

Surgery is definitive mode of treatment because there is lack of spontaneous regression, a high rate of recurrent infection, the possibility of other diagnoses, and rare malignant degeneration. Acute inflammation is treated medically unless incision and drainage or aspiration of an abscess is required. Three to four weeks should pass after an acute infection before a definitive surgical exploration is undertaken. In our series 60% of patients took medical treatment and 3.33% underwent drainage of abscess before definitive surgical excision of the lesion. Surgical excision of lesion was done in all patients.

### 4.5. Complications

In the series by Ford et al. [3], there was a postoperative recurrence rate of 3%. In our series recurrence rate was 1.2%. It is our observation that while methylene blue dye enhances visualization of the larger and more proximal (in relation to the punctum) part of the tracts and ramifications, it does not demarcate the most peripheral ramifications and hence a conscious attempt must be made to follow the tracts till the end. Using magnification loops or the microscope at the time of dissection may enhance prospects of complete removal. While it may be impossible to guarantee a complete removal, injection of dye and microscopic removal may be vital in preventing recurrences. We believe that this along with the practice of excision of the tract along with surrounding tissue has led to a low recurrence rate seen in our series. In our series, postoperative wound infection was the most common postoperative complication seen in 14.71% patients. This relatively high incidence can be attributed to the fact that a high percentage of the patients in this series were from a low socioeconomic stratum. 8.82% had wound gaping requiring secondary suturing. Though facial nerve paralysis/weakness has been reported in patients undergoing superficial parotidectomy for first branchial cleft anomalies, none of our patients had involvement of facial nerve.

## 5. Individual Branchial Anomalies

### 5.1. First Branchial Cleft Anomalies

First branchial cleft anomalies are thought to develop as a result of incomplete obliteration of the cleft between the mandibular process of the first arch and the second arch. A sinus will have an opening in the upper neck or in the floor of the external auditory canal, and a fistula will have an opening in both of these sites. The first branchial cleft anomalies have been classified as Type I or Type II by work [6].

Type I is considered to be a duplication of cartilaginous external auditory canal. A cystic mass in the postauricular area extends medially and anteriorly along the external auditory canal. It usually passes lateral to the facial nerve and ends at the bony meatus. No external opening is present except after infection. Type II is considered to be a duplication of the cartilaginous external auditory canal and pinna. A sinus passes from an external opening high in the neck along the anterior border of SCM muscle, superficial or deep to the facial nerve in close relation to the parotid gland. It can either end blindly at the floor of the cartilaginous external auditory canal or open in to the external auditory canal, which is called the collaural fistula. In both types entrapment of desquamating squamous epithelium will result in the production of a cholesteatoma process resulting in erosion of bony external meatus, tympanic annulus, and hypotympanum [7].

In the series by Belenky and Medina [8] 66.66% of patients belonged to Work I and 33.33% to Work II. In the study by Nofsinger et al. [9] 27.27% had Work I and 72.73% had Work II lesions. In our study, Work I constituted 17.65% and Work II constituted 20.59%. In the study by Triglia et al. [10] on the first branchial cleft anomalies, 30.77% were male and 69.23% female. In the study by Belenky and Medina [8] the incidence in male was 22.22% and in female was 77.77%. In our study, 17.65% were male and 20.59% were female. All these studies show a higher incidence of first branchial cleft among female. The symptoms and signs related to these anomalies in this series are similar to those described by various authors. In general, both types of anomalies may present as a progressively enlarging or recurrent mass or as a draining sinus. The diagnosis is usually made after infection has taken place. Incision and drainage of an abscess are frequently needed before definitive surgical treatment can be performed. Histopathology of the Work I lesions in our study showed that 100% of cases lined by squamous epithelium and 20% had cartilage components in the subepithelial layer. In the study by Belenky and Medina [8] 16.7% of Work I had cartilage component in the subepithelium. Intraoperatively the lesion was found superficial to the facial nerve in 100% of our cases. Belenky and Medina [8] reported that the lesion was superficial to facial nerve in 88.88% of cases. In the study by Triglia et al. [10] lesion was deep to facial nerve in 39% cases. In the series by Nofsinger et al. [9], the lesion was deep to the facial nerve in 55% of cases. We agree with Bajaj et al. [4, 11] that it is advisable to perform a superficial parotidectomy in cases of first cleft anomalies while identifying the tract in relation to the facial nerve.

### 5.2. Second Branchial Cleft/Pouch Anomalies

During embryonic development, the second arch grows caudally, enveloping the third, fourth, and sixth arches and fusing with skin caudal to these arches, forming a deep groove (cervical sinus). The edges of this grove then meet and fuse. The ectoderm within the fused tube then disappears. Persistence of the ectoderm gives rise to a branchial cyst. A branchial fistula results from the breakdown of the endoderm. A persistent fistula of the second branchial cleft and pouch usually has its external opening in the neck near mid or lower part of SCM muscle. As it ascends it pierces platysma. At the level of hyoid it curves medially and passes between the external and internal carotids in relation to the hypoglossal and glossopharyngeal nerves. It opens in to the oropharynx usually in the intratonsillar cleft of palatine tonsil. In series of 98 cases by Ford et al. [3] 78% presented by the age of five years and in vast majority there was history of intermittent discharge and infection of neck sinus since birth. In seven percent there was history of incision and drainage of an associated neck abscess. In his series 60% sinus opening was on the right side and 40% on left. In our series of 17 cases 70% presented at age above 11 years. Only one patient presented at birth. 64.7% occurred on the right side and 35.3% on the left.

**Figure 7:**
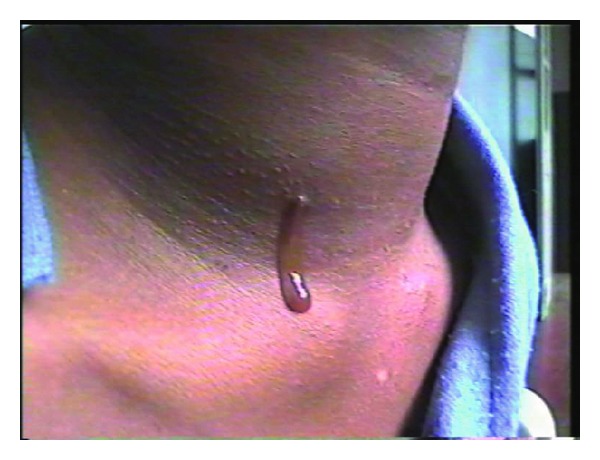
Saliva coming out of third branchial fistula.

Second arch anomalies may take several forms. There may be only a simple sinus opening that extends up the neck for a variable distance. Branchial fistulas commonly present with persistent mucoid discharge from an opening in the skin of the neck. But rare and unusual presentation have also been documented. They have been documented as to present as parapharyngeal mass located in the supratonsillar fossa and extending to the lateral nasopharynx [12]. Exceedingly rarely, a branchial cleft anomaly may be found to be malignant on presentation [13]. A complete branchial fistula with external and internal opening is rare. The completeness of a fistula is diagnosed by a dye test in which methylene blue is injected through the outer opening and appears in the throat. A negative preoperative outcome on the test might become positive under general anaesthesia because of muscle relaxation. Occasionally, the fistula tract may be blocked by secretion or granulation giving negative fistula test [14]. In many a case, saliva is seen dribbling from the neck opening, which itself proves the completeness of the tract (Figure 7). In the 62 pediatric second branchial cleft anomalies, Bajaj et al. [4] reported 50 of them to be unilateral and 12 to be bilateral.

Several surgical approaches have been described for the management of a branchial fistula. The stepladder approach [15] was described in 1933. The fistulous tract can be approached through a series of stepladder incision first encompassing the sinus opening and second overlying the carotid bifurcation. Subsequently the parapharyngeal portion of the fistula can be approached perorally after tonsillectomy. A wide cervicotomy incision (hockey stick) [16] can also be used which allows for adequate exposure of neck structure for accurate dissection. In all our cases, we traced the fistula up to the tonsillar area and excised the tract.

### 5.3. Third and Fourth Branchial Cleft/Pouch Anomalies

Third branchial anomalies are rare and constitute less than 1% of all such cases. Here the fistula opening is seen in the lower neck and it passes along the carotid sheath and then passes between the glossopharyngeal and hypoglossal nerve, piercing the thyrohyoid membrane to enter pharynx in the region of pyriform fossa. Anomalies of this type are very rare. Third pouch remnants are described as passing superior to superior laryngeal nerve and posterior to the common carotid artery. The tract emerges above the thyroid cartilage.

A persistent fistula of the fourth branchial cleft and pouch is theoretically possible but is very rare. Here the fistula opens in to the lower part of neck near the SCM muscle. The tract then passes inferiorly between superior and recurrent laryngeal nerve into the mediastinum, looping around the arch of aorta in the left and subclavian artery in the right and passes back in to the neck, ascending posterior to the carotid. Then it passes between the thyroid and cricoid cartilages and opens in to the pyriform fossa. According to Godin et al. [17], almost all the fourth arch anomalies reported occurred in the left side. In our series also, the fourth arch fistula occurred in the left side.

Third and fourth branchial remnants have been reported at any age. In neonates, these anomalies can be dangerous because of rapid enlargement leading to tracheal compression and respiratory distress. Noncommunicating or noninfected communicating cysts may present as cold thyroid nodules [18]. When infected, diagnosis and successful excision of a pyriform fossa sinus are very challenging and require meticulous approach. A history of recurrent upper respiratory tract infection, neck or thyroid pain and tenderness, and neck mass is common. Other presentations include cellulites, hoarseness, odynophagia, thyroiditis, abscess, and stridor. A combination of ultrasound and CT with or without oral contrast will assist in the diagnosis.

We had two cases each of third and fourth branchial anomalies. All these patients came to us with after recurrences following surgeries done elsewhere. In cases of fourth cleft anomalies, CT with contrast through the neck opening demonstrated dye in the mediastinum. The third branchial anomalies showed a fistulous opening in the lower neck, which, on dissection, passed between the glossopharyngeal and hypoglossal nerve as classically described, piercing the thyrohyoid membrane to enter pharynx in the region of pyriform fossa. In the cases of fourth branchial anomalies, the intramediastinal part of the fistula was left behind with their ends ligated and the rest of the tract was dissected out from the superior mediastinum up to the pyriform fossa.

## 6. Highlights

1. Our series confirms a higher incidence of first branchial cleft anomaly among females, the cause of which needs to be investigated.
2. None of our patients had involvement of facial nerve. All patients had tracts going superficial to the facial nerve.
3. All the fourth arch anomalies in our series occurred on the left side which is consistent with the literature.
4. While it may be impossible to guarantee a complete removal, injection of dye and microscopic removal may be vital in preventing recurrences. We believe that this along with the practice of excision of the tract along with surrounding tissue has led to a low recurrence rate seen in our series.

## 7. Conclusion

Branchial apparatus plays an important role in the development of head and neck structures. Aberrant development of these structures can lead to formation of different anomalies. Most of these anomalies remain asymptomatic and might present later in life. Diagnosis is rather easy with a proper knowledge of the anatomy of the branchial anomalies. Confirming the extent of the tract is mandatory before any surgery as these lesions pass in relation to some of the most vital structures of the neck. Surgery must always be the treatment option for these lesions due to the fact that these lesions do not regress spontaneously and they have a high incidence of recurrent infection. Surgery also gives a chance to diagnose by means of histopathology, the rare occurence of branchogenic carcinoma.

## Figures and Tables

**Table 1:** Incidence of individual anomalies.

Branchial arch involved	Cyst		Fistula		Total	
	No.	%	No.	%	No.	%
1st branchial arch (*n* = 13) (38.24%)						
Work I	6	17.65%			6	17.65%
Work II			7	20.59%	7	20.59%
2nd branchial arch (*n* = 17) (50%)						
Branchial cyst	8	23.53%			8	23.53%
Branchial fistula			9	26.47%	9	26.47%
3rd branchial arch (*n* = 2) (5.88%)	—	—	2	5.88%	2	5.88%
4th branchial arch (*n* = 2) (5.88%)	—		2	5.88%	2	5.88%

Total (*n* = 34)	14	41.18%	20	58.82%	34	100%

**Table 2:** Age and sex incidence.

Age in years	1st arch				2nd arch		3rd arch		4th arch	
	Work I		Work II							
	No.	%	No.	%	No.	%	No.	%	No.	%
0–5										
M	—	—	—	—	—	—	—	—	—	—
F	—	—	—	—	1	2.96%	—	—	—	—
6–10										
M	—	—	1	2.94%	2	5.88%	—	—	—	—
F	—	—	3	8.82%	2	5.88%	—	—	—	—
11–20										
M	2	5.88%	—	—	4	11.76%	—	—	1	2.94%
F	1	2.94%	2	5.88%	3	8.82%	—	—	—	—
21–40										
M	2	5.88%			3	8.82%	1	2.94%	1	2.94%
F	1	2.94%	—	—	2	5.88%	—	—	—	—
≥41										
M	—	—	—	—	—	—	1	2.94%	—	—
F	—	—	—	—	—	—	—	—	—	—

Total (*n* = 34)										
M	4	11.76%	2	5.88%	9	26.47%	2	2.94%	2	2.94%
F	2	5.88%	5	14.70%	8	23.53%	—	—	—	—

**Table 3:** Clinical features.

Clinical features	1st arch		2nd arch		3rd arch		4th arch		Total	
	No.	%	No.	%	No.	%	No.	%	No.	%
Swelling										
Neck	3	8.82%	9	26.47%	1	2.94%	1	2.94%	14	41.18%
Postauricular	7	20.59%	—	—	—	—	—	—	7	20.59%
Sinus										
Neck	8	23.53%	8	23.53%	1	2.94%	1	2.94%	18	52.94%
Pain	5	14.71%	3	8.82%	—	—	1	2.94%	9	26.47%
Fever	2	5.88%	3	8.82%	—	—		—	5	14.71%
Discharge	5	14.71%	7	20.59%	1	2.94%	1	2.94%	14	41.18%

**Table 4:** Treatment.

	1st arch		2nd arch		3rd arch		4th arch		Total	
	No.	%	No.	%	No.	%	No.	%	No.	%
Antibiotics	12	40%	4	13.33%	1	3.33%	1	3.33%	18	60%
I and D	1	3.33%	—	—	—	—	—		1	3.33%
Excision										
(i) Single incision	13	43.33%	7	23.33%	1	3.33%	1	3.33%	22	73.33%
(ii) Stepladder incision	—	—	7	23.33%	—	—	—		7	23.33%

**Table 5:** Complications.

	1st arch		2nd arch		3rd arch		4th arch		Total	
	No.	%	No.	%	No.	%	No.	%	No.	%
Wound infection	4	11.76%	1	2.94%	—	—	—	—	5	14.71%
Wound gaping	2	5.88%	1	2.94%	—	—	—	—	3	8.82%
Neurological deficit	—	—	1	2.94%	—	—	—	—	1	2.94%
Dermatitis	1	2.94%	—	—	—	—	—	—	1	2.94%

Total (34)	7	20.59%	3	8.82%	—	—	—	—	10	29.41%
